# Sexual dysfunction among Egyptian men with chronic hepatitis C in the post elimination era prevalence and associated factors

**DOI:** 10.1038/s41598-026-37013-z

**Published:** 2026-02-19

**Authors:** Ammal M. Metwally, Mahi M Al-Tehewy, Nihad A. Ibrahim, Walaa A. Fouad, Hanan S. Ezelarab, Runia F. El-Folly, Ahmed M. Omar, Ehab Kamal, Hazem M. El-Hariri

**Affiliations:** 1 Public Health and Community Medicine Research Department, Medical Research and Clinical Studies Institute, National Research Centre, P.O: 12622, Dokki, 60014618 Giza Egypt; 2 Community Medicine Department, Faculty of Medicine, Ain Shams University, Cairo, Egypt; 3 Tropical Medicine Department, Faculty of Medicine, Ain Shams University, Cairo, Egypt; 4 Dermatology and Andrology Department, Medical Research and Clinical Studies Institute, National Research Centre, P.O: 12622, Dokki, 60014618 Giza Egypt; 5 Complementary Medicine Department, Medical Research and Clinical Studies Institute, National Research Centre, P.O: 12622, Dokki, 60014618 Giza Egypt

**Keywords:** Hepatitis c virus (HCV), Sexual dysfunction, Erectile dysfunction, Liver disease severity, Post-Elimination era, Egypt, Diseases, Medical research, Risk factors

## Abstract

Despite Egypt’s landmark achievement in controlling hepatitis C virus (HCV) and receiving WHO validation on the path to elimination, long-term sequelae among men previously affected by chronic HCV remain under-recognized. Sexual dysfunction is an important yet often overlooked component of survivorship and quality of life in chronic liver disease care. To estimate the prevalence and identify factors independently associated with sexual dysfunction among Egyptian men with chronic HCV, informing post-elimination care strategies. A cross-sectional analysis was conducted on 1,500 adult males attending National Committee for Control of Viral Hepatitis (NCCVH) units across six geographically diverse Egyptian governorates. Sexual health was assessed using the Brief Sexual Symptom Checklist for Men and the International Index of Erectile Function (IIEF-5). Logistic regression was applied to identify factors independently associated with sexual dysfunction. The overall prevalence of at least one form of sexual dysfunction was 72.8%. Erectile dysfunction was most common (59.3%), followed by desire dysfunction (47.5%) and premature ejaculation (32.1%), orgasmic dysfunction (27.8%), and dyspareunia (12.6%). The strongest independent associations clustered around markers of advanced disease severity and complications, particularly hepatocellular carcinoma (DD AOR: 12.39; 95% CI: 4.64–33.09; PE AOR: 8.99; 95% CI: 2.06–39.25) and age ≥ 50 years (DD AOR: 5.00; 95% CI: 3.65–6.85; PE AOR: 7.58; 95% CI: 4.17–13.76). Diabetes mellitus was also strongly associated with erectile dysfunction (AOR: 3.89; 95% CI: 2.07–7.30) and premature ejaculation (AOR: 3.33; 95% CI: 1.72–6.44). Sexual dysfunction is a common, under-recognized sequela among Egyptian men with chronic HCV in the post-elimination era. The strongest independent associations were observed with advanced liver disease/complications and metabolic comorbidity, supporting integration of routine sexual health assessment and counseling into post-HCV follow-up. Survivorship-oriented care is particularly important for patients with advanced liver disease.

## Introduction

Chronic hepatitis C remains a significant global cause of chronic liver disease, contributing to liver fibrosis, cirrhosis, and hepatocellular carcinoma^1^. An estimated 58 million individuals live with chronic HCV worldwide, accounting for approximately 290,000 deaths annually^2^. Since the introduction of oral direct-acting antivirals (DAAs) around 2015, the global HCV care cascade has improved, although challenges persist across regions^3^. Egypt has made remarkable progress through a national elimination program, achieving HCV antibody prevalence below 1% and 93% treatment coverage among diagnosed individuals, supporting WHO verification efforts^4^.

Despite these advances, many individuals continue to experience long-term consequences of chronic HCV. Social isolation, anxiety, and psychological distress are common and may be exacerbated by stigma related to misconceptions about sexual transmission^5–7^. Sexual health is a key component of quality of life^8^, and sexual dysfunction (SD); disruption in one or more phases of the male sexual response cycle is frequently reported in chronic liver disease^8–12^. SD generally worsens with disease progression and cirrhosis, potentially reflecting declining testosterone levels^13^, hepatic insufficiency, and medication effects^10^. Proposed mechanisms in chronic HCV include hypothalamic-pituitary-gonadal axis disruption^11^, viremia, and detection of viral particles in semen with possible sperm damage^14^. More broadly, SD in chronic liver disease plausibly reflects overlapping endocrine and vascular pathways, with additional contributions from inflammation, sarcopenia/physical deconditioning, and psychological comorbidity^15^. Neuropsychiatric sequelae in HCV; fatigue, depression, cognitive and stigma-related distress may further impair sexual symptoms and quality of life; although SVR is generally associated with improved HRQoL, symptom burden may persist in some patients^16,17^.

Male SD includes multiple domains with variable prevalence across populations^14^. Desire dysfunction may affect up to 25% of men, erectile dysfunction affects approximately 31.9% of men aged 40–80 years, and premature ejaculation occurs in about 3.7% to 24.9% across age groups^14^.

In Egypt, research on sexual health outcomes among men affected by HCV remains limited^18^. Few studies have assessed multiple SD domains in relation to liver disease severity, and none has provided a comprehensive multicenter national estimate^12,19^.

Unlike many prior single-center reports, the present national multicenter analysis evaluates SD across Child–Pugh grades in the post-elimination era and examines clinical, behavioral, and medication exposures within a unified analytic framework^20–22^. Given the DAA scale-up and the evolving clinical profile of the HCV population, assessing lingering sequelae; including SD and their impact on well-being remains a priority^23–25^, particularly in settings where health literacy, sexual-health stigma, and access to services vary^17,26^.

While Egypt has achieved major milestones toward HCV elimination, many previously infected individuals continue to live with advanced liver disease. In men with chronic HCV, SD may contribute to reduced quality of life, psychological distress, and relationship strain, and may persist in those with established cirrhosis despite virologic cure^1,7,8,16,17,27,28^.

The aim of this study was twofold. First, it sought to estimate the prevalence of various sexual dysfunction domains among Egyptian male patients with chronic hepatitis C attending selected treatment centers in six governorates representing different regions of Egypt. Second, it aimed to explore associated factors of sexual dysfunction in this population, including disease severity, complications, and selected medication exposures commonly used in chronic liver disease care (e.g., beta-blockers, spironolactone, and tramadol), in order to guide early identification, targeted interventions, and improved quality of life outcomes.

## Methodology

### Study Design, Setting, and participants

This cross-sectional study was conducted on 1,500 Egyptian male patients diagnosed with chronic hepatitis C virus (HCV) infection. The participants were recruited from the National Committee of Control of Viral Hepatitis (NCCVH) treatment and follow-up units over a period of two years (January 2021 to January 2023). These NCCVH units operate within governmental hospital settings and serve as specialized hepatitis care services, including secondary and referral-level facilities across participating governorates.

### Inclusion and exclusion criteria

Eligible participants were males who had reached puberty, were younger than 60 years, diagnosed with chronic HCV liver disease, currently married and living with a sexually active spouse, and willing to participate in the study. This criterion was used to ensure that sexual function outcomes were assessed among men with recent partnered sexual activity; however, it may limit generalizability to unmarried men, men without current partners, and older age groups. Patients were excluded if they exhibited impaired awareness or consciousness, had a history of sexual dysfunction prior to HCV diagnosis, or suffered sexual dysfunction due to trauma, surgeries, or other direct causes unrelated to chronic HCV that may affect genital nerve supply.

### Sample size

The required sample size was estimated based on a reported prevalence of sexual dysfunction among HCV-positive patients of 53.6%^27^. Using the PASS software (11th release), a sample of 1,500 was calculated to provide sufficient statistical power at a 95% confidence level with a margin of error of 0.05.

### Sampling technique

A multistage random sampling technique was employed. The sampling frame included twelve Ministry of Health treatment units across six governorates, selected to represent Egypt’s four main regions: Cairo (Urban Governorates); Dakahleya and Gharbia (Lower Egypt); Beni-Suef and Assiut (Upper Egypt); and Ismailia (Canal region). The proportion of participants from each governorate was based on the regional prevalence of HCV antibodies: Cairo (12.0%), Dakahleya (17.7%), Gharbia (20.9%), Beni-Suef (10.1%), Assiut (15.2%), and Ismailia (24.1%)^18^. Further stratification within each treatment unit was aligned with national frequencies of Child-Pugh liver disease severity: Child-A (14.3%), Child-B (33.3%), and Child-C (52.4%)^12,19^.

### Tools and data collection

Data were collected through structured interviews, including demographic characteristics, lifestyle habits over the previous six months, HCV diagnosis and treatment history, comorbidities, and medication use. Lifestyle habits included smoking status and regular tea consumption as recorded during interview. Medication history captured the use of agents with potential sexual function effects (e.g., tramadol, beta-blockers, and spironolactone), recorded as current use (yes/no). Sexual function was assessed using two validated tools: the Brief Sexual Symptom Checklist for Men (BSSC-M)^29^ and the International Index of Erectile Function-5 (IIEF-5)^30^. The BSSC-M was employed to screen for types of sexual dysfunction in primary care settings^31^. The IIEF-5 is a five-item instrument that evaluates the ability to achieve and maintain an erection during sexual activity, with established psychometric properties including high internal consistency (0.73–0.95), test-retest reliability (0.64–0.84), sensitivity (0.98), and specificity (0.88). The IIEF-5 score ranges from 5 to 25 and was categorized as severe (5–7), moderate (8–11), mild-to-moderate (12–16), mild (17–21), and no dysfunction (22–25). Internal consistency of the administered tools was evaluated using Cronbach’s alpha (α) to assess reliability in the current sample^30,32^.

Liver disease severity was assessed using the Child-Pugh classification system^29^, which evaluates five clinical and laboratory parameters: total bilirubin, serum albumin, prothrombin time or INR, ascites, and hepatic encephalopathy. For total bilirubin, a level of less than 2.0 mg/dL is assigned 1 point, between 2.0 and 3.0 mg/dL is given 2 points, and more than 3.0 mg/dL corresponds to 3 points. Serum albumin levels above 3.5 g/dL receive 1 point, levels between 2.8 and 3.5 g/dL are scored 2 points, and levels below 2.8 g/dL are scored 3 points. Regarding coagulation, a prothrombin time of less than 4.0 s or an INR below 1.7 is scored as 1 point, a prothrombin time between 4.0 and 6.0 s or INR from 1.7 to 2.3 receives 2 points, and values above 6.0 s or INR over 2.3 receive 3 points. For ascites, the absence of ascites is scored as 1 point, mild ascites as 2 points, and moderate to severe ascites as 3 points. Hepatic encephalopathy is graded similarly: none corresponds to 1 point, grade I–II to 2 points, and grade III–IV to 3 points.

### Implementation to ensure validity and reliability

The Arabic versions of both tools were produced through translation and back-translation. Initial translations were done into Arabic, followed by reverse translation into English by an independent researcher to ensure content equivalence and linguistic validity.

### Study dependent variables

The domains of sexual dysfunction were defined according to Yafi et al.^14^. These included desire dysfunction (reduced or absent sexual interest), erectile dysfunction (inability to achieve or maintain an erection adequate for sexual activity), PE (ejaculation within one minute of vaginal penetration with inability to delay), orgasmic dysfunction or delayed ejaculation (marked delay or reduced intensity of orgasm), and dyspareunia (genital pain during intercourse).

### Statistical analysis

Data were reviewed for completeness and consistency before being coded, tabulated, and analyzed using IBM SPSS Statistics version 24.0^33^. Descriptive statistics were used to summarize the data, with means and standard deviations for continuous variables and frequencies and percentages for categorical variables.

Age-adjusted frequencies of sexual dysfunction were calculated using the direct adjustment method, applying the age-specific dysfunction rates of Child-B and Child-C patients to the corresponding age strata of the Child-A group^34^.

Associations between continuous independent variables (e.g., age) and sexual dysfunction outcomes were evaluated using one-way analysis of variance (ANOVA), followed by Bonferroni post hoc correction for multiple comparisons. Prior to conducting ANOVA, assumptions of normality and homogeneity of variances were assessed using the Shapiro-Wilk test and Levene’s test, respectively.

For categorical independent variables (e.g., education level, occupation, residence), the Chi-square test was used to assess differences in proportions across severity groups. When expected cell counts were less than 5, Fisher’s exact test was employed to ensure accuracy. For comparisons across ordered liver severity categories (Child-Pugh classes), trend was evaluated using the linear-by-linear association (Chi-square test for trend), with Bonferroni-adjusted post hoc comparisons where applicable as specified in table footnotes.

To determine independent associated factors of sexual dysfunction, binary logistic regression analysis was performed. Variables with a p-value < 0.05 in bivariate analysis were included in the multivariable model. In addition, clinically important variables (e.g., age and liver disease severity) and medication exposures with biologic plausibility were retained to reduce residual confounding even if borderline in bivariate testing. The results were presented as odds ratios (OR) with 95% confidence intervals (CI). Model fit was evaluated using the Hosmer-Lemeshow goodness-of-fit test, and multicollinearity was assessed using the variance inflation factor (VIF). To reduce model instability, events-per-variable (EPV) was checked (target EPV ≥ 10), and categorical coding (e.g., Child-Pugh class and age groups) was verified prior to modelling. When quasi-complete separation or extreme estimates were suspected, penalized logistic regression (Firth correction) was considered as a sensitivity approach to obtain finite, more stable estimates. Model discrimination was evaluated using the area under the ROC curve (AUC), alongside calibration (Hosmer–Lemeshow). All tests were two-sided. A p-value < 0.05 was considered statistically significant throughout the analysis.

## Results

HCV chronic male patients (1500 cases) were classified into 215 (14.3%) child-A, 500 (33.3%) child-B and 785 (52.4%) child-C.

### Socio-Demographic and clinical characteristics by HCV severity

Table 1 shows that the mean age of patients significantly increased with disease severity, from 30.4 ± 6.3 years in Child-A to 49.5 ± 4.2 years in Child-C (*p* < 0.001). A clear inverse relationship was observed between educational attainment and liver disease severity: illiteracy and basic education levels were most prevalent in Child-C patients (32.8% and 38.9%, respectively), while higher education was most frequent among Child-A patients (37.2%). Additionally, manual occupations, including drivers, farmers, and laborers, were significantly more common in Child-C (45.3%) than Child-A (11.2%). Rural residence also became more frequent with worsening liver function, increasing from 44.0% in Child-A to 56.5% in Child-C (*p* = 0.014). Moreover, the duration since diagnosis and the mean age at diagnosis rose progressively with disease advancement, indicating delayed recognition and progression of liver pathology.

**Table 1:** socio-demographics and clinical data according to HCV disease severity.

Variables		All cases (*N* = 1500)	Child-A (*N* = 215)	Child-B (*N* = 500)	Child-C (*N* = 785)	*P*
**Age in years ( mean** ± SD**)**		44.8 ± 7.9	30.4 ± 5.3α	43.6 ± 5.2β	49.5 ± 3.8ω	^<0.001*
**Age categories** **(years)**	**20.0–**	108 (7.2%)	104(48.4%)α	4 (0.8%) β	0 (0.0%) ω	#<0.001*
	**30.0–**	214 (14.3%)	102 (47.4%)	108(21.6%)	4 (0.5%)	
	**40.0–**	717 (47.8%)	6 (2.8%)	319(63.8%)	392 (49.9%)	
	**50.0–59.0**	461 (30.7%)	3 (1.4%)	69 (13.8%)	389 (49.6%)	
**Number of** **current wives**	**One wife**	1452(96.8%)	209 (97.2%)	489(97.8%)	754 (96.1%)	#0.170
	**more than one wife**	48 (3.2%)	6 (2.8%)	11 (2.2%)	31 (3.9%)	
**Marriage duration (years)**		18.3 ± 7.7	8.0 ± 4.2 α	16.1 ± 5.3 β	22.6 ± 6.2 ω	^<0.001*
**Youngest wife`s age (years)**		40.8 ± 7.5	28.3 ± 4.8 α	39.2 ± 4.6 β	45.2 ± 4.9 ω	^<0.001*
**Governorate**	**Cairo**	180 (12.0%)	25 (11.6%)	60 (12.0%)	95 (12.1%)	**#**0.942
	**Dakahlia**	267 (17.8%)	39 (18.1%)	89 (17.8%)	139 (17.7%)	
	**Gharbia**	313 (20.9%)	45 (20.9%)	104(20.8%)	164 (20.9%)	
	**Ismailiah**	152 (10.1%)	22 (10.2%)	50 (10.0%)	80 (10.2%)	
	**Assuit**	227 (15.1%)	32 (14.9%)	76 (15.2%)	119 (15.2%)	
	**Beni-Suif**	361 (24.1%)	52 (24.2%)	121(24.2%)	188 (23.9%)	
**Residence**	**Urban**	796 (53.1%)	121 (56.3%)	271(54.2%)	404 (51.5%)	**#**0.163
	**Rural**	704 (46.9%)	94 (43.7%)	229(45.8%)	381 (48.5%)	
**Education**	**Illiterate**	186 (12.4%)	14 (6.5%) α	33 (6.6%) β	139(17.7%)ω	#<0.001*
	**R& W**	253 (16.9%)	14 (6.5%)	55 (11.0%)	184 (23.4%)	
	**Primary**	189 (12.6%)	16 (7.4%)	63 (12.6%)	110 (14.0%)	
	**Preparatory**	391 (26.1%)	30 (14.0%)	140(28.0%)	221 (28.2%)	
	**Secondary**	161 (10.7%)	67 (31.2%)	74 (14.8%)	20 (2.5%)	
	**Higher**	320 (21.3%)	74 (34.4%)	135(27.0%)	111 (14.1%)	
**Occupation**	**Not working**	65 (4.3%)	9 (4.2%) α	18 (3.6%) β	38 (4.8%) ω	#<0.001*
	**Manual**	470 (31.3%)	44 (20.5%)	117(23.4%)	309 (39.4%)	
	**Clerks**	935 (62.3%)	156 (72.6%)	354(70.8%)	425 (54.1%)	
	**Professional**	30 (2.0%)	6 (2.8%)	11 (2.2%)	13 (1.7%)	
**Age at diagnosis (years)**		**39.3 ± 7.9**	**25.6 ± 4.8 α**	**37.9 ± 5.5 β**	**44.0 ± 4.4 ω**	^<0.001*
**Duration since diagnosis (years)**		**5.4 ± 2.1**	**4.8 ± 2.2 α**	**5.6 ± 2.0 β**	**5.5 ± 2.0 β**	^<0.001*

Percentages were per columns. Acute: Acute HCV manifestations. Chronic: Chronic HCV manifestations, Accidental: accidental discovery during routine lab testing. ^ANOVA test with post hoc Bonferroni test. #Linear by linear association with post hoc Bonferroni test. Homogenous groups had the same letter” α, β, ω”. *Significant.

### Sexual dysfunction domains by HCV severity

Table 2 shows that the prevalence of all major domains of sexual dysfunction, desire dysfunction, erectile dysfunction, and PE rose significantly dramatically with the severity of liver disease. DD increased from 4.7% in Child-A to 98.1% in Child-C, ED from 6.0% to 100%, and PE from 9.3% to 100%, (*p* < 0.001).

**Table 2:** Sexual dysfunction domains according to HCV disease severity as assessed by the brief sexual symptom check list for men (BSSC-M).

Sexual dysfunction		All cases (*N* = 1500)	Child-A (*N* = 215)	Child-B (*N* = 500)	Child-C (*N* = 785)	*P*
**Desire dysfunction** 95% CI		849(56.6%) 54.1%-59.1%	10 (4.7%) α 1.8%–7.5%	69 (13.8%) β 10.8%–16.8%	770 (98.1%) ω 97.1%–99.0%	#<0.001*
**Erectile dysfunction** 95% CI		931(62.1%) 59.6%-64.5%	13 (6.0%) α 2.9%–9.2%	133 (26.6%) β 22.7%–30.5%	785 (100%) ω 100.0%–100.0	#<0.001*
**Premature** **ejaculation (PE)**		1002(66.8%)	20 (9.3%) α	197 (39.4%) β	785 (100%) ω	#<0.001*
95% CI		64.4%-69.2%	5.4%–13.2%	35.1%–43.7%	100.0%–100.0%	
**^Common presenting complains**	**Erection**	929 (92.3%)	11 (50.0%)	133 (66.5%)	785 (100%)	#<0.001*
	**Premature Ejaculation**	72 (7.1%)	8 (36.4%)	64 (32.0%)	0 (0.0%)	
	**Desire**	6 (0.6%)	3 (13.6%) α	3 (1.5%) β	0 (0.0%) ω	

Percentages are column percentages within each Child-Pugh class. Denominators (N) are provided in column headers; prevalence estimates are accompanied by 95% CIs. #P values correspond to the Chi-square test for trend (linear-by-linear association) across ordered Child-Pugh categories (A→B→C). When an overall trend test was significant, pairwise post hoc comparisons between Child-Pugh classes were conducted with Bonferroni adjustment. Groups sharing the same letter (α/β/ω) are not significantly different. Significant at *p* < 0.05.

### Age-adjusted sexual dysfunction by HCV severity

None of the studied cases complained of delayed ejaculation, dyspareunia, or abnormal flexures. Table 3*shows* that even after adjusting for age, the increasing trend in dysfunctions remained significant. The adjusted prevalence of DD, ED, and PE in Child-C remained 100%, whereas it was significantly lower in Child-A (4.8%, 6.1%, and 10.0%, respectively; *p* < 0.001). Meanwhile, PE percentages were significantly highest in child-C followed by child-B and lowest in child-A with a significant difference between all child grades.

**Table 3:** Age adjusted sexual dysfunctions as assessed by the brief sexual symptom check list for men (BSSC-M) according to HCV disease severity.

Dysfunction	Child-A (*N* = 215)	Child-B (*N* = 215)	Child-C (*N* = 111)	*P*
**Desire** 95% CI	10 (4.7%) α 1.8%–7.5%	16 (7.4%) α 3.9%–11.0%	111 (100%) β 100.0%–100.0%	**#** < 0.001*
**Erection** 95% CI	13 (6.0%) α 2.9%–9.2%	21 (9.8%) α 5.8%–13.7%	111 (100%) β 100.0%–100.0%	**#** < 0.001*
**Premature** **ejaculation (PE)** 95% CI	20 (9.3%) α 5.4%–13.2%	83 (38.6%) β 32.1%–45.1%	111 (100%) ω 100.0%–100.0%	**#** < 0.001*

Denominators (N) are provided in column headers; Percentages are column percentages within each Child-Pugh group after age standardization. Age-adjusted estimates were obtained by direct age standardization, using the Child-A age distribution as the standard (reference) population; Child-B and Child-C age-specific rates were weighted to match Child-A. # Linear-by-linear association (trend) test with Bonferroni-adjusted post hoc comparisons. Groups sharing the same letter (α/β/ω) are not significantly different. *Significant at p* < 0.05.

### Erectile dysfunction severity by child grade

**Fig. 1:**
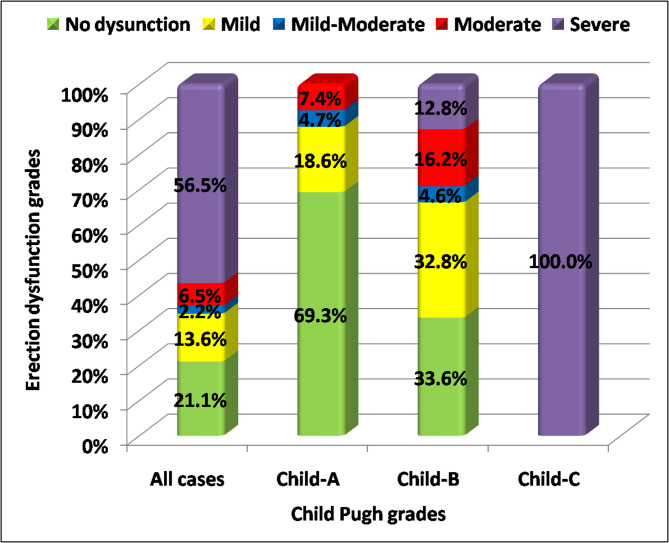
Erection dysfunction grades according to HCV disease severity. Percentages were per columns. *Figure depicts severity categories based on IIEF-5 scoring across Child-Pugh classes.*

Figure 1 shows that the severity of erectile dysfunction escalates with liver disease grade. Figure 1 complements Tables 2 and 3 by presenting the distribution of IIEF-5 erectile dysfunction severity categories across Child-Pugh classes (i.e., severity staging rather than presence/absence alone). Mild dysfunction was predominant in Child-A, and severe dysfunction occurred exclusively in Child-C patients (100%) (p < 0.001).

### Demographic factors associated with sexual dysfunction domains

Table 4 shows that older age (≥ 40 years) was significantly associated with ED (66%) and PE (57%) (p < 0.001). Basic education or illiteracy was reported in 41.5% of ED and 45.5% of PE patients, while those without dysfunction had significantly higher educational levels (p < 0.001). Dysfunction was more frequent among patients with more than one wife (p < 0.05). While, residence and occupation, did not show statistically significant differences across dysfunction domains. The duration since diagnosis and age at diagnosis, were significantly more frequent in dysfunction cases (p < 0.01).

**Table 4:** Demographic and clinical characteristics according to sexual dysfunction domains.

Variables		PE (*N* = 1002)		Erectile Dysf. (*N* = 931)	Desire Dysf. (*N* = 849)	*P*
**Age (years)**		48.4 ± 4.9		48.7 ± 4.6	48.9 ± 4.6	^0.04*
**Age grades (years)**	**20.0–**	2 (1.9%)		2 (1.9%)	107 (99.1%)	< 0.001**
	**30.0–**	47 (22.0%)		24 (11.2%)	183 (85.5%)	
	**40.0–**	515 (71.8%)		426 (59.4%)	246 (34.3%)	
	**50.0–59.0**	438 (95.0%)		397 (86.1%)	33 (7.2%)	
**Number of** **current wives**	**one wife**	965 (66.5%)		819 (56.4%)	553 (38.1%)	0.64
	**more than one wife**	37 (77.1%)		30 (62.5%)	16 (33.3%)	
**Marriage duration (years)**		21.4 ± 6.6		21.8 ± 6.5	22.0 ± 6.5	^**0.20**
**Youngest wife age (years)**		44.1 ± 5.5		44.4 ± 5.4	44.6 ± 5.4	^**0.39**
**Governorate**	**Cairo**	111 (61.7%)		97 (53.9%)	77 (42.8%)	0.97
	**Dakahlia**	180 (67.4%)		156 (58.4%)	97 (36.3%)	
	**Gharbia**	208 (66.5%)		174 (55.6%)	118 (37.7%)	
	**Ismailia**	106 (69.7%)		91 (59.9%)	55 (36.2%)	
	**Assiut**	157 (69.2%)		126 (55.5%)	85 (37.4%)	
	**Beni Suef**	240 (66.5%)		205 (56.8%)	137 (38.0%)	
**Residence**	**Urban**	518 (65.1%)		438 (55.0%)	314 (39.4%)	0.34
	**Rural**	484 (68.8%)		411 (58.4%)	255 (36.2%)	
**Education**	**Illiterate**	160 (86.0%)		141 (75.8%)	31 (16.7%)	0.018*
	**R& W**	211 (83.4%)		192 (75.9%)	52 (20.6%)	
	**Primary**	142 (75.1%)		120 (63.5%)	54 (28.6%)	
	**Preparatory**	270 (69.1%)		229 (58.6%)	140 (35.8%)	
	**Secondary**	47 (29.2%)		32 (19.9%)	123 (76.4%)	
	**High**	172 (53.8%)		135 (42.2%)	169 (52.8%)	
**Occupation**	**Not working**	47 (72.3%)		41 (63.1%)	21 (32.3%)	**0.86**
	**Manual**	366 (77.9%)		324 (68.9%)	122 (26.0%)	
	**Official**	571 (61.1%)		470 (50.3%)	411 (44.0%)	
	**Professional**	18 (60.0%)		14 (46.7%)	15 (50.0%)	
**Age at diagnosis (years)**		**42.8 ± 5.4**		**43.2 ± 5.2**	**43.3 ± 5.2**	^**0.99**
**Duration since diagnosis (years)**		**5.5 ± 2.0**		**5.5 ± 2.0**	**5.6 ± 2.0**	^**0.65**

Denominators (N) are provided in column headers, ^ANOVA test with post hoc Bonferroni test. *Significant, ** highly Significant.

### Univariate associations of lifestyle/medication exposures and key comorbidities with sexual dysfunction domains

Table 5 shows the univariate distribution of lifestyle/medication exposures (tea, tramadol, beta-blockers, spironolactone) and key comorbidities (diabetes mellitus and hepatocellular carcinoma) by sexual dysfunction domains among men with chronic hepatitis C. Tea consumption was high and comparable across all domains, with no significant differences (P = 0.946 for DD, P = 0.922 for ED, and P = 0.762 for PE). Tramadol intake differed significantly across domains (all P < 0.001). Beta-blocker use was significantly more frequent in ED and PE (P < 0.001), whereas the association with DD was not significant (P = 0.725). Spironolactone use was markedly more frequent in DD (770/849; 90.7%), followed by ED (782/931; 84.0%) and PE (782/1002; 78.0%), with significant differences across all domains (all P < 0.001). Diabetes mellitus and hepatocellular carcinoma were also significantly distributed across domains (all P < 0.001), with DM reported in 13.8–15.6% and HCC in 8.4–9.5% of participants across the respective dysfunction domains.

**Table 5:** Univariate distribution of lifestyle/medication exposures and key comorbidities by sexual dysfunction domains among men with chronic hepatitis C.

Variable	Premature ejaculation (*N* = 1002) *n* (%)	Erectile dysfunction (*N* = 931) *n* (%)	Desire dysfunction (*N* = 849) *n* (%)
Tea consumption: Yes No	924 (92.2%) 78 (7.8%)	858 (92.2%) 73 (7.8%)	782 (92.1%) 67 (7.9%)
**P value**	0.762	0.922	0.946
Tramadol intake, Yes No	47 (4.7%) 955 (95.3%)	37 (4.0%) 894 (96.0%)	25 (2.9%) 824 (97.1%)
**P value**	< 0.001**	< 0.001**	< 0.001**
Beta-blocker use Yes No	149 (14.9%) 853 (85.1%)	137 (14.7%) 794 (85.3%)	98 (11.5%) 751 (88.5%)
**P value**	< 0.001**	< 0.001**	0.725
Spironolactone use Yes No	782 (78.0%) 220 (22.0%)	782 (84.0%) 149 (16.0%)	770 (90.7%) 79 (9.3%)
**P value**	< 0.001**	< 0.001**	< 0.001**
Diabetes mellitus (DM) Yes No	155 (15.5%) 847 (84.5%)	145 (15.6%) 786 (84.4%)	117 (13.8%) 732 (86.2%)
**P value**	< 0.001**	< 0.001**	< 0.001**
Hepatocellular carcinoma (HCC) Yes No	84 (8.4%) 918 (91.6%)	83 (8.9%) 848 (91.1%)	81 (9.5%) 768 (90.5%)
**P value**	< 0.001**	< 0.001**	< 0.001**

Denominators (N) are provided in column headers. Values are n (%); percentages are **per column**. P values compare exposure (Yes/No) across each outcome column using **χ²** (or **Fisher’s exact** when expected counts < 5). Two-sided *p* < 0.05.; *Significant at p  < 0.05*, ** highly Significant.

### Associated factors with sexual dysfunction

Table 6 presents multivariable logistic regression models showing that different factors were independently associated with specific sexual dysfunction domains. For desire dysfunction, hepatocellular carcinoma (HCC) showed a strong positive association (AOR: 12.391; 95% CI: 4.639–33.093), age ≥ 50 years (AOR: 5.003; 95% CI: 3.652–6.854) and higher Child score (per unit increase) (AOR: 1.028; 95% CI: 1.013–1.044), while tramadol intake showed an inverse association (AOR: 0.106; 95% CI: 0.060–0.187).

For erectile dysfunction, spironolactone use showed a very large association (AOR: 1001.04; 95% CI: 240.459–4167.435), with higher odds also observed for age ≥ 50 years (AOR: 5.210; 95% CI: 3.063–8.863), diabetes mellitus (AOR: 3.885; 95% CI: 2.069–7.295), beta-blocker use (AOR: 2.929; 95% CI: 1.706–5.028), and higher Child score (AOR: 1.085; 95% CI: 1.007–1.170), whereas tea consumption showed an inverse association (AOR: 0.082; 95% CI: 0.046–0.144). Given the very large spironolactone AOR, the magnitude of this estimate should be interpreted cautiously as it may reflect sparse data or quasi-complete separation; sensitivity analysis using penalized logistic regression (Firth correction) was considered as described in Statistical Analysis.

For PE, significant positive associations were observed for HCC (AOR: 8.994; 95% CI: 2.061–39.247), age ≥ 50 years (AOR: 7.577; 95% CI: 4.171–13.763), diabetes mellitus (AOR: 3.332; 95% CI: 1.724–6.440), increasing Child score (AOR: 1.780; 95% CI: 1.614–1.962), beta-blocker use (AOR: 1.760; 95% CI: 1.045–2.962), and age ≥ 40 years (AOR: 1.700; 95% CI: 1.128–2.562), while tramadol intake (AOR: 0.450; 95% CI: 0.251–0.807) and tea consumption (AOR: 0.007; 95% CI: 0.003–0.014) showed inverse associations.

**Table 6:** Logistic regression for factors affecting SD domains among chronic HCV cases.

Factors	β	SE	*P*	AOR (95% CI)
Desire Dysfunction (DD)				
**Hepatocellular carcinoma (HCC)**	2.517	0.501	< 0.001*	12.391 (4.639–33.093)
**Age ≥ 50.0**	1.610	0.161	< 0.001*	5.003 (3.652–6.854)
**Child score**	0.028	0.008	< 0.001*	1.028 (1.013–1.044)
**Tramadol**	-2.242	0.288	< 0.001*	0.106 (0.060–0.187)
**Erectile Dysfunction (ED)**				
**Spironolactone**	6.909	0.728	< 0.001*	1001.04 (240.459-4167.435)
**Age ≥ 50.0**	1.651	0.271	< 0.001*	5.210 (3.063–8.863)
**Diabetes mellitus (DM)**	1.357	0.321	< 0.001*	3.885 (2.069–7.295)
**Beta-blocker**	1.075	0.276	< 0.001*	2.929 (1.706–5.028)
**Child score**	0.082	0.038	0.032*	1.085 (1.007–1.170)
**Tea**	-2.507	0.291	< 0.001*	0.082 (0.046–0.144)
**Premature Ejaculation (PE)**				
**HCC**	2.197	0.752	0.003*	8.994 (2.061–39.247)
**Age ≥ 50.0**	2.025	0.305	< 0.001*	7.577 (4.171–13.763)
**DM**	1.204	0.336	< 0.001*	3.332 (1.724–6.440)
**Child score**	0.576	0.050	< 0.001*	1.780 (1.614–1.962)
**Beta-blocker**	0.565	0.266	0.033*	1.760 (1.045–2.962)
**Age 40–49 years**	0.530	0.209	0.011*	1.700 (1.128–2.562)
**Tramadol**	-0.798	0.298	0.007*	0.450 (0.251–0.807)
**Tea**	-4.975	0.359	< 0.001*	0.007 (0.003–0.014)

β: Regression coefficient. SE: Standard error. AOR: Adjusted Odds ratio. CI: Confidence interval. HCC: hepatocellular carcinoma; DM: diabetes mellitus. *Significant at *p* < 0.05.

## Discussion

Sexual dysfunction (SD) is common and affects multiple phases of male sexual function^9^, with erectile dysfunction closely linked to reduced relationship satisfaction^11^. Chronic HCV has been associated with a high SD burden, often intersecting with depressive symptoms, underscoring the importance of psychosocial assessment^28^. In Egypt’s post-elimination context^4^, long-term sequelae remain clinically important. This national multicenter cross-sectional study quantified SD prevalence and examined factors associated with SD domains among 1,500 men with chronic HCV across Child–Pugh stages.

Sociodemographic gradients were evident, with more advanced liver disease clustering with older age and indicators of lower socioeconomic position, consistent with delayed presentation and access barriers reported in Egypt^35,36^, particularly in rural settings^37,38^. Early HCV is often asymptomatic and may be detected incidentally^39,40^, whereas advanced disease may present later with symptoms/complications as patients age^41^ and as health-care seeking increases with disease burden^42,43^.

Across domains, SD increased sharply with liver disease progression (Tables 2 and 3), with very high rates in Child-C that likely reflect the burden of advanced cirrhosis in this treatment-center cohort; however, the cross-sectional design does not allow etiologic separation of HCV-specific effects from cirrhosis severity per se. These rates exceed those reported in the general population^14^ and are consistent with prior studies in cirrhosis and chronic hepatitis^11,44,45^. Mechanistically, hepatic decompensation is linked to hormonal disturbance (including reduced testosterone and HPG-axis disruption)^46^, and cirrhosis-related fatigue, mood disturbance, vascular dysfunction, and metabolic changes may further exacerbate SD^19^. High SD burden is repeatedly reported in advanced liver disease, particularly Child–Pugh B/C^47^. Similar dysfunction is reported in cirrhosis from other etiologies, with shared endocrine and vascular contributors^12,15,20^. In chronic HCV, extrahepatic manifestations and stigma-related psychological distress may further compound symptoms, but severity of decompensation appears to be a major driver across etiologies^17,21,25^. Consistent with prior work, cirrhotic men may experience reduced desire, confidence, and satisfaction^8^, with androgen deficiency as a plausible mediator (free testosterone can be informative)^13^. Age-adjusted analyses in our cohort continued to show the highest burden in Child-C, suggesting differences are not explained by age alone; systemic inflammation and fatigue may also contribute^48–50^, consistent with observations from other settings^8,51^.

Lower educational attainment was associated with higher SD prevalence, suggesting a potential role for health literacy and care engagement^52,53^. Although other sociodemographic variables were not consistently significant, trends by rural residence/manual labor may reflect broader access disparities^37,42,54^.

In multivariable models, several clinical and behavioral factors were independently associated with SD domains. Advanced disease markers (higher Child score and HCC) and older age (≥ 50 years) showed strong associations, aligning with cumulative systemic effects of decompensation and reinforcing the survivorship relevance in the post-elimination era^19,20,47^. Associations were also observed for diabetes and commonly used medications^16,20,21^, and diabetes and HCC have been linked to worse erectile/sexual outcomes in other work^55,56^. In the DAA era, SVR is generally associated with improved HRQoL, yet patients with established cirrhosis may continue to experience symptom burden; integrating structured sexual health assessment and counseling into post-SVR follow-up may therefore be particularly relevant for men with advanced fibrosis/cirrhosis^16,20,28,57^.

Medication/lifestyle signals require cautious interpretation. Tramadol showed inverse associations in some models, which is biologically plausible for premature ejaculation given serotonergic/noradrenergic effects and supportive RCT/meta-analytic evidence for on-demand use^58–60^; however, tramadol carries dependence risk^61–63^, and opioid exposure and tramadol misuse/dependence have been linked to hypogonadism, erectile dysfunction, and reduced libido^64–67^. Accordingly, these findings should be interpreted as associations that may reflect confounding by indication, reverse causality, or reporting/coding differences rather than causal benefit. For tea, short-term vascular/endothelial plausibility exists^68,69^, but population evidence is mixed and in our dataset univariate tea exposure was high and comparable across outcome groups (Table 5), supporting cautious interpretation of adjusted associations. Tea is therefore presented as hypothesis-generating, requiring confirmation with detailed exposure quantification and sensitivity analyses^68–73^.

### Implications

The high SD burden—particularly with advancing disease—supports integrating routine sexual health screening, counseling, and referral pathways into chronic liver disease follow-up. Behavioral risk contexts remain relevant in Egypt^74,75^, and community-based approaches have been effective across related health priorities, including viral hepatitis^76–78^, diabetes^79^, renal disease^80^, and maternal/child health^81–83^. These findings support a shift from elimination-only metrics toward comprehensive post-eradication survivorship care.

### Strengths of the study

This study has several notable strengths. It is among the largest and most comprehensive investigations of sexual dysfunction among male patients with chronic hepatitis C in Egypt, incorporating a multicenter sample across six governorates that represent diverse geographic and socioeconomic settings. The use of validated tools, such as the Brief Sexual Symptom Checklist for Men (BSSC-M) and the International Index of Erectile Function (IIEF-5), adds methodological rigor. The stratification by Child-Pugh classification allowed for meaningful comparisons across liver disease severity stages, and the multivariate analysis identified independent associated factors while adjusting for confounders. Furthermore, age-adjusted analyses enhanced the validity of group comparisons.

### Limitation of the study

However, some limitations should be acknowledged. The cross-sectional design precludes causal inferences and limits the ability to assess changes in sexual function over time or following treatment. The reliance on self-reported data for sexual function may introduce reporting bias, particularly in a conservative cultural context where stigma may influence disclosure. Recruitment through specialized NCCVH hospital-based units may introduce referral bias, enriching the sample with more severe cases. PE was assessed using the BSSC-M as a pragmatic symptom checklist; a dedicated instrument such as the Premature Ejaculation Diagnostic Tool (PEDT) could provide more granular and standardized classification in future studies. The exclusion of unmarried or sexually inactive men introduces potential selection bias and may limit generalizability to unmarried men, men without current partners, sexually inactive men, and older age groups (≥ 60 years). In addition, potential confounders such as psychiatric comorbidities or partner-related factors were not assessed. We also did not measure hormonal profiles (e.g., total/free testosterone), which may mediate sexual dysfunction in chronic liver disease. Finally, while associations with medications such as tramadol and tea were observed, these findings should be interpreted with caution due to the lack of pharmacological detail and potential for reverse causality. Moreover, because the study did not include a comparator group with non-HCV cirrhosis, we could not directly contrast sexual dysfunction attributable to cirrhosis severity versus cirrhosis etiology. In addition, laboratory screening for sexually transmitted infections (e.g., syphilis) was not included in the protocol; therefore, undiagnosed STIs could not be evaluated as potential contributors to sexual symptoms, and future studies should consider incorporating STI assessment where feasible.

### Conclusion and future directions

In the wake of Egypt’s progress toward hepatitis C elimination, this national multicenter study brings critical attention to a persistent and under-recognized burden: sexual dysfunction among male survivors of chronic HCV. Our findings reveal a high prevalence of sexual dysfunction, with advancing liver disease, especially Child-Pugh Class C emerging as the strongest associated factor. Sociodemographic disparities, including lower educational attainment, rural residence, and manual labor occupations, were significantly associated with worse sexual health outcomes, highlighting inequities in health literacy and care access. Comorbid conditions such as diabetes and hepatocellular carcinoma, as well as certain medication exposures, also played a significant role.

Interestingly, the observed inverse associations with tramadol use and tea consumption merit cautious interpretation, as they may reflect confounding or unmeasured behavioral factors rather than causal relationships. These signals nonetheless point to the need for further mechanistic and behavioral research.

In the post-elimination era, addressing the long-term quality of life for individuals with prior HCV infection is no longer optional but essential. Integrating routine screening, patient-centered counseling, and culturally sensitive management of sexual dysfunction into liver disease care pathways will be vital to sustaining the public health gains of Egypt’s HCV response.

Future research should focus on: longitudinal tracking of sexual function after viral clearance to inform recovery trajectories; interventional studies assessing the effectiveness of pharmacologic, behavioral, and educational strategies to improve sexual health in this population; and qualitative assessments of patient and provider attitudes, barriers, and facilitators to open discussions around sexual health in the context of chronic liver disease care. In addition, multi-country studies and collaborations with centers outside Egypt are needed to assess the generalizability of these findings across different populations and health-system contexts.

## Data Availability

The datasets generated and/or analysed during the current study are not publicly available due to ethical restrictions involving human subjects and the inclusion of sensitive health-related information that could potentially compromise participant privacy and confidentiality. Additionally, the informed consent obtained from participants did not include provisions for public data sharing. Furthermore, parts of the dataset are being used in ongoing related analyses that are intended for future publication. However, the data are available from the corresponding author upon reasonable request and subject to institutional and ethical approvals.

## Abbreviations

ANOVA: Analysis of Variance
BSSC: M–Brief Sexual Symptom Checklist for Men
CI: Confidence Interval
DAAs: Direct–Acting Antivirals
ED: Erectile Dysfunction
HCC: Hepatocellular Carcinoma
HCV: Hepatitis C Virus
IIEF: 5–International Index of Erectile Function–5 Items
NCCVH: National Committee for Control of Viral Hepatitis
PE: Premature Ejaculation
PEDT: Premature Ejaculation Diagnostic Tool
SD: Sexual Dysfunction
SPSS: Statistical Package for the Social Sciences
VIF: Variance Inflation Factor
WHO: World Health Organization
